# The Wine of Circe

**DOI:** 10.3201/eid3104.AC3104

**Published:** 2025-04

**Authors:** Terence Chorba

**Affiliations:** Centers for Disease Control and Prevention, Atlanta, Georgia, USA

**Keywords:** The Wine of Circe, art–science connection, foodborne illness, food safety

**Figure:**
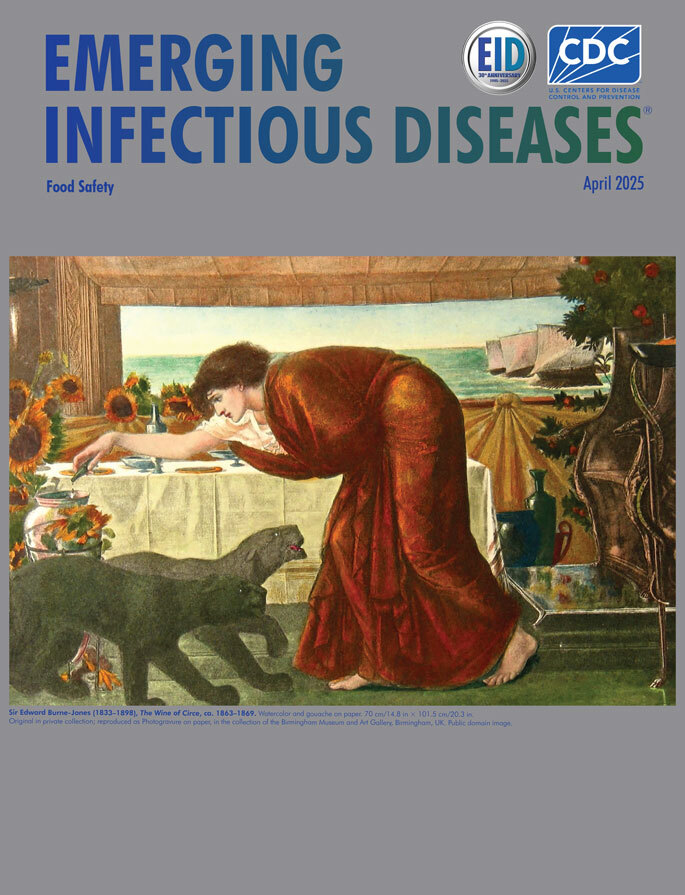
Sir Edward Burne-Jones (1833–1898). ***The Wine of Circe***, ca. 1863–1869. Watercolor and gouache on paper. 70 cm/14.8 in × 101.5 cm/20.3 in. Original in private collection; reproduced as Photogravure on paper, in the collection of the Birmingham Museum and Art Gallery, Birmingham, UK. Public domain image.

As a minor deity in ancient Greek mythology of the 8th–6th Century BCE, Circe (Κίρκη) occupies a poorly understood place in the Homeric tradition (*1*). She is frequently confused with the nymph Calypso because of parallels in their unpredictable behaviors and in their associations with Odysseus, as described in *The Odyssey* of Homer (*2*). Circe is a master of medicinal preparations and herbs, and Homer repeatedly accords her the epithet polypharmakos (πολυφαρμάκος), “knowing many drugs” (*3*). Using potions together with her magical wand, she wields the power to change visitors or adversaries into animals such as pigs, lions, or wolves.

Circe’s renowned legend appears in books 10 and 12 of *The Odyssey*, where Odysseus visits her island, Aeaea, during his journey home from the Trojan War (*4*). She hosts his crew with a seemingly benign feast featuring kykeon (κυκεών)—an Ancient Greece drink commonly comprising a mixture of goat’s cheese, barley meal, and wine—to which Circe then adds honey. She then laces the concoction with a potion that induces amnesia and hallucinations and turns its partakers into pigs, all in the interest of preemptively neutralizing any threat from these foreign visitors and of getting Odysseus to remain with her on her lonely island. Forewarned by Hermes, the divine messenger, Odysseus avoids this fate by chewing a magical herb, moly (μῶλυ), which Hermes has provided to him in advance, as a prophylactic antidote to Circe’s potion. Protected from Circe’s enchantments, Odysseus then convinces her to restore his crew to their human forms.

This month’s cover of *Emerging Infectious Diseases* features a watercolor and gouache depiction of Circe by Sir Edward Burne-Jones (1833–1898). A later figure in the 19th Century Pre-Raphaelite movement in England, and a master of arts and crafts and of decorative design, Burne-Jones often portrayed mythological and medieval themes, both in his watercolors and in oils (Figure) (*5*,*6*). In the cover piece, Circe looms large, her commanding presence spanning the painting as she leans toward a pot or jug, preparing a potion. At her feet, 2 panther-like beasts, likely transformed humans, reflect and accentuate her predatory nature. Through the window behind her, several sailing ships approach the island, presumably bearing future subjects of her sorcery, including Odysseus and his crew, destined to encounter her cunning magic.

**Figure:**
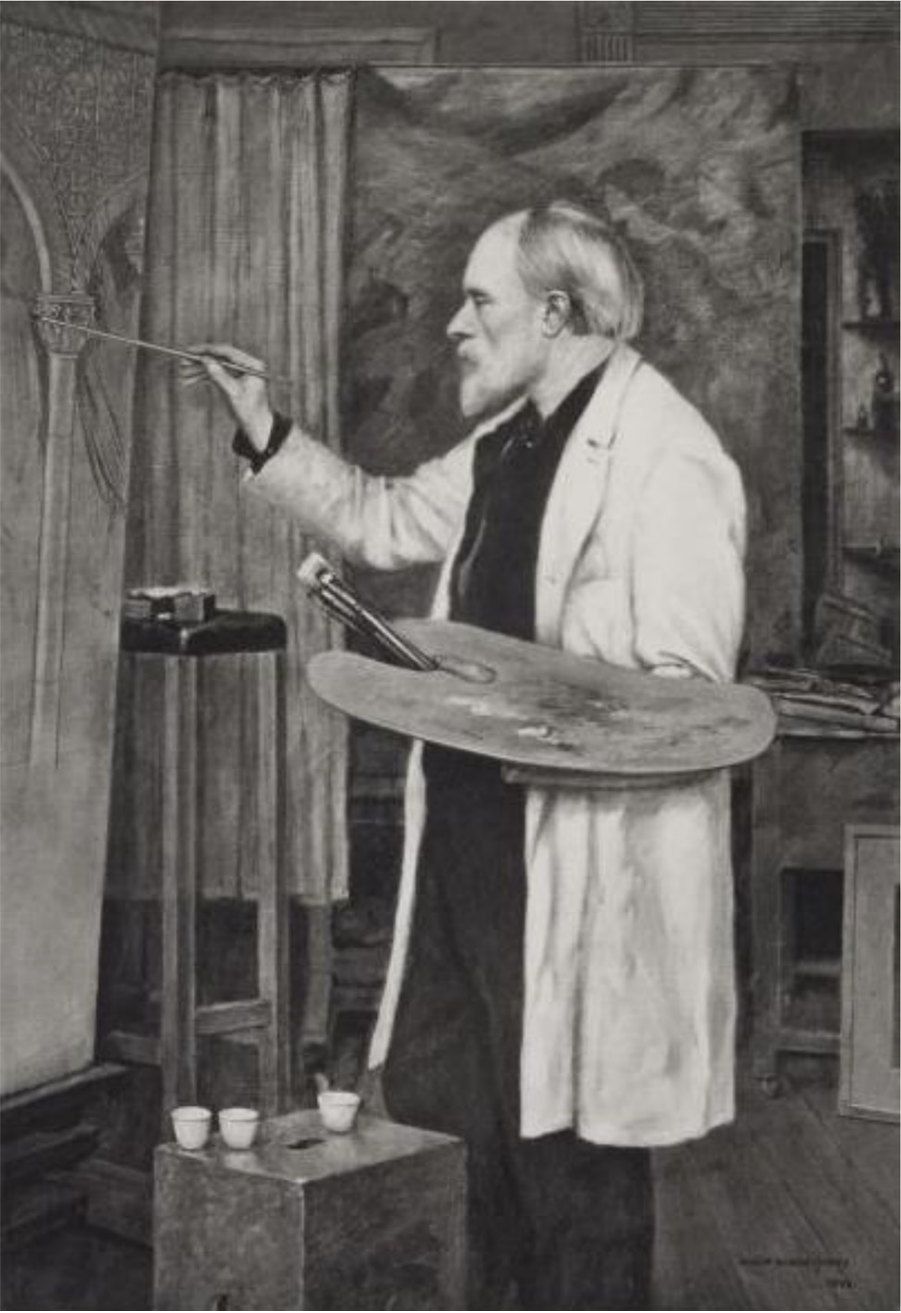
Photogravure (ca. 1900) by Frederick Hollyer of a portrait of Edward Burne-Jones painting, by his son, Philip Burne-Jones, 1898. Source: Birmingham Museum and Art Gallery, Birmingham, UK.

The association of wine as a medium for intentional poisoning extends beyond mythology and into historical accounts. In the 4th Century BCE in Athens, Plato famously immortalized Socrates’ death, the result of drinking hemlock-laced wine and subsequently experiencing its effects of respiratory paralysis and muscular spasms (*7*). As a physician and pharmacologist in the 1st Century CE in the Emperor Nero’s court, Dioscorides described the sinister properties of arsenic—lacking color, odor, or taste when mixed with food or drink; used as a poison, arsenic readily causes abdominal distress, confusion, paralysis, and death. Although Homer does not specify the agent that Circe used, scholars have hypothesized datura (jimsonweed), a nightshade that contains a variety of deliriants and anticholinergic alkaloids, including scopolamine and atropine, known since antiquity for inducing amnesia, hallucinations, and inhibition of the parasympathetic nervous system. If so, the herb moly could correspond to the common snowdrop (*Galanthus nivalis*), a bulbous, perennial European plant which contains galantamine, a compound used to counter anticholinergic poisoning (*8*).

The enduring themes of Circe’s legend underscore the universal human tendency to interpret natural phenomena through myth. The poisoning of Odysseus’s crew can be framed as a foodborne illness outbreak. Such illnesses usually arise from consuming food or beverages that have been unintentionally contaminated by any of a variety of bacteria, viruses, and parasites (*9*). However, intentional food contamination, from introduction of microorganisms, toxins, chemicals, or contaminants, has also been the subject of numerous epidemiologic investigations (*10*–*15*). Toxins implicated in foodborne illness are usually the result of growth of toxin-producing organisms, most commonly *Bacillus cereus*, *Staphylococcus aureus*, *Clostridium botulinum*, or *Clostridium perfringens*.

Today, foodborne illnesses remain a significant public health challenge. In the United States, 7 major pathogens caused an estimated 9.9 million cases of foodborne illness in 2019 and led to an estimated 53,300 hospitalizations and 931 deaths (*16*). The most common causes of domestically acquired foodborne illness were norovirus (5.5 million), *Campylobacter* (1.9 million), *Salmonella* (1.3 million), *Clostridium perfringens* (889,000), and Shiga toxin–producing *Escherichia coli* (357,000). The most common causes of death from domestically acquired foodborne illness were *Salmonella*, *Campylobacter*, norovirus, and invasive *Listeria monocytogenes*. The tallies of foodborne illness presented in this issue highlight the ongoing complexity and scope of foodborne diseases.

As Western poets and artists continue to draw inspiration from Greek mythology, the stories of figures like Circe retain their relevance. From ancient legends of transformation and poisoning to modern challenges of food safety, Circe’s tale of foodborne illness, albeit intentional, reminds us of the enduring interplay between myth, art, and human experience.
